# Introducing a Remote Patient Monitoring Usability Impact Model to Overcome Challenges

**DOI:** 10.3390/s24123977

**Published:** 2024-06-19

**Authors:** Steffen Baumann, Richard T. Stone, Esraa Abdelall

**Affiliations:** 1 Department of Industrial and Manufacturing Systems Engineering, Iowa State University, Ames, IA 50011, USA; rstone@iastate.edu; 2 Department of Industrial Engineering, Jordan University of Science and Technology, Ar-Ramtha 3030, Jordan; abdelallesra@gmail.com

**Keywords:** remote patient monitoring, telehealth, virtual care, RPM Usability Impact model, user-centered design, usability, wearables, home-use medical devices

## Abstract

Telehealth and remote patient monitoring (RPM), in particular, have been through a massive surge of adoption since 2020. This initiative has proven potential for the patient and the healthcare provider in areas such as reductions in the cost of care. While home-use medical devices or wearables have been shown to be beneficial, a literature review illustrates challenges with the data generated, driven by limited device usability. This could lead to inaccurate data when an exam is completed without clinical supervision, with the consequence that incorrect data lead to improper treatment. Upon further analysis of the existing literature, the RPM Usability Impact model is introduced. The goal is to guide researchers and device manufacturers to increase the usability of wearable and home-use medical devices in the future. The importance of this model is highlighted when the user-centered design process is integrated, which is needed to develop these types of devices to provide the proper user experience.

## 1. Introduction

### 1.1. Wearables and Home-Use Medical Devices

Patient-generated health data (PGHD) devices, including home-use medical devices, wearables, as well as other consumer health and fitness trackers, have become a major part of our lives. The research firm Grand View Research reports that the medical wearables market was valued in 2020 at USD 16.6 billion [1], and a survey conducted in 2019 reported that 38% of Americans used some kind of fitness tracker in the past [2].

The Healthcare Information and Management Systems Society (HIMSS) conducted a Fitbit-sponsored survey amongst 101 healthcare professionals (decision makers in IT, administrators, and clinicians), reporting that 79% of respondents agreed that more data in between patient appointments allow clinicians to receive a more holistic view of a patient’s health with the possibility to intervene early. The HIMSS further highlights that 72% of all respondents agreed that these data are vital to providing better patient care [3].

### 1.2. The State of the Remote Patient Monitoring (RPM) Program in a Changing Healthcare System

The most recent developments in the healthcare field are largely driven by the COVID-19 pandemic and provide further opportunities for telehealth, as many patients in rural areas have been encouraged to not leave their homes amid the outbreak of the virus [4]. According to Bestsennyy et al. [5], up to 24% of all visits to a healthcare facility can be held virtually, supplementing digital health with office visits to improve patient care.

Remote patient monitoring (RPM) is a sub-category of telehealth [6]. It enables providers to detect a patient’s physiological deterioration and obtain more detailed health status information to make treatment decisions [7]. RPM is defined by The National Telehealth Policy Center as “collection, storage, and evaluation of health information (patient’s vital signs, blood sugar levels, etc.) through live monitoring via devices that transmit information from the home or care facility to a provider” [8]. One of the key concepts of RPM is monitoring chronic conditions in the areas of respiratory conditions, weight management, and cardiovascular management [9,10,11,12,13]. In total, 67% of Medicare recipients suffer under a minimum of two such chronic conditions [7], and the National Center for Chronic Disease Prevention and Health Promotion (CDC) reports that 90% of U.S. healthcare expenditure is used to treat patients with such conditions [14], underlining the impact RPM can have on the population as well as healthcare expenditures in the U.S.

To assess the current state and trends of RPM, the authors of this manuscript reviewed data points and trends and identified that PRM experienced a significant growth in research publications, particularly driven by the need to address hospital capacity issues during the COVID-19 pandemic. For example, there was an increase in monthly remote monitoring claims from 91 to 594 per 100,000 enrollees between February 2020 and September 2021 [15]. Mueller [16] adds that RPM services have been predominantly provided by primary care cardiology and pulmonary specialties during the pandemic.

Before COVID-19, funding and adoption for telehealth and remote patient monitoring were relatively modest. For instance, the Telehealth Network Grant Program (TNGP) awarded around USD 8.7 million annually for telehealth technologies in rural and medically underserved areas. Additionally, the Telehealth Resource Center (TRC) Grant Program was funded at approximately USD 4.6 million per year from 2017 [17]. In response to COVID-19, the CARES Act significantly increased telehealth funding, allocating USD 29 million annually for five years to the TNGP [17]. The consumer adoption of telehealth services surged from 11% pre-pandemic to 46% during the pandemic, and the telehealth market potential grew to an estimated USD 250 billion of current U.S. healthcare spending that could be virtualized [4]. These changes reflect a major shift in both the funding landscape and the utilization of telehealth and RPM technologies, illustrating how the pandemic has driven rapid transformation and integration of digital health solutions into mainstream healthcare practices.

Reported studies report that RPM is beneficial for both patients and providers [18,19], and RPM has shown the potential to lower the massive expenditure that burdens the healthcare system, and with that, there are lower emergency department visits, hospitalizations, and 30-day readmissions rates of up to 72% [20]. Trinity Health, for instance, a Catholic health system in Michigan, reported a decrease in 30-day readmission rates from 16% to 6% within one year [21].

In addition, a changing healthcare system that is navigating toward a new payment system called value-based care that enables providers to receive reimbursements based on the patient’s health outcome [22] prompted the Center for Medicare & Medicaid Services (CMS) in 2019 to add four more CPT codes, which are 99453, 99454, 99457 and 99458 [7,23,24]. This extends the reimbursement to initial device setup, remotely reviewing the collected data, including reimbursement for supplying devices, training patients on device usage, and generating data on a monthly basis, as well as an additional 20 min within the same month for an additional session needed for the patient or patient’s caregiver [23]. For these to be applied, there should be a minimum of 16 days’ worth of data collected within one calendar month [25].

The adoption of RPM has grown and has become more accepted, predominantly due to the COVID-19 pandemic. According to [5], 11% of U.S. consumers used some type of telehealth service pre-COVID-19; however, during COVID-19, the number rose to 46%, and according to Strategic Market Research [26], physicians’ perception of remote patient monitoring initiatives grew from 87% in 2016 to 95% in 2022, with the actual growth of RPM adoption by physicians ranging from 14% in 2016 to 80% in 2022. It is estimated that by 2025, 26% of the U.S. population will use some type of RPM device.

Also, all patients who benefit from consistent monitoring (patients with acute or chronic conditions, post-operative care after hospital discharge) are eligible to receive the RPM service as long as needed, with the requirement for this to occur while the patient is under the supervision of a qualified physician [27,28].

### 1.3. IoT-Enabled Consumer-Grade Wearables vs. IoT-Enabled Home-Use Medical Devices

Commonly used PGHD devices to monitor a patient’s health during an RPM program are blood pressure monitors, pulse oximeters, heart rate monitors, glucometers, thermometers, scales, or respiratory monitors [29]. Many times, these devices are web-enabled or Internet-of-Things (IoT)-enabled computers and sensors worn on the patient’s body. These devices capture a variety of patient’s physiological parameters, such as sleep quality, respiratory rate, or heart rate, to provide insights into potential health conditions, such as sleep apnea or hypertension [30,31].

In order for the collected data to be useful, it needs to be ensured that the collected data are accurate and precise and, at the same time, do not cause harm to the patient, as unsafe operation and inaccurate data are driven by limited patient usability. The term usability is defined by the International Organization for Standardization (ISO) 9241-11 as ”the extent to which a product can be used by specified users to achieve specified goals with effectiveness, efficiency, and satisfaction in a specified context of use” [32].

The usability and user experience of a PGHD device are, however, impacted by the user’s capabilities, which are limited many times due to accessibility constraints (such as hand tremors, vision or hearing disabilities), health literacy, or limited technical skills, hence creating challenges for safe operation and accurate data acquisition [33,34,35,36,37]. The usability of medical devices, including home-use devices, can significantly impact the accuracy of results and data interpretation, as usability issues can lead to user errors during device setup, calibration, or data collection. For example, if a blood glucose monitor has a complex interface, users may input incorrect information, affecting the accuracy of readings [38,39]. The operation of such devices in RPM or virtual health can, therefore, cause inadequate data generation, which may, in turn, impact the way a patient is treated.

**Figure 1:**
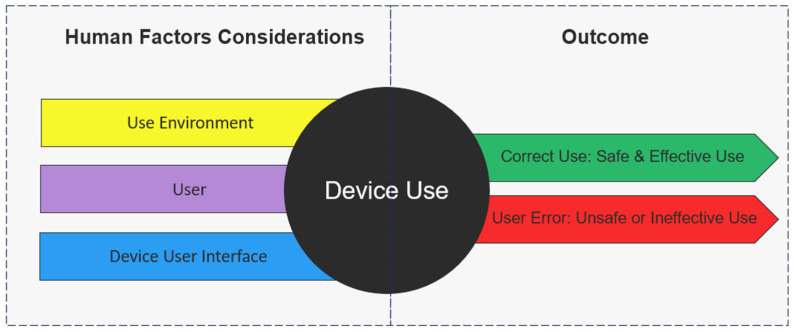
Impact of proper device usage based on human factors. Adapted from Applying human factors and usability engineering to medical devices: guidance for industry and Food and Drug Administration staff (Food and Drug Administration, 2016).

According to the Food and Drug Administration (FDA) [40] and as illustrated in Figure 1, usability deficiencies for medical devices can be traced back to device users (the persona using the product, such as a clinician, a caregiver, or the patient), device use environments (location the device is intended to be used, such as healthcare facility or home), and device user interfaces (components of the device that interface with the user, such as the display, a membrane switch, batteries, a charger or a label). The FDA refers to this as “Human Factor Considerations”. This guidance document assists industry professionals in following appropriate human factors and usability engineering processes with the goal of maximizing device usability, ensuring new medical devices will be safe and effective for their intended users, uses, and use environments.

## 2. Previous Work

The FDA’s guidance document ensures that medical devices are designed and optimized for use in the specific environments where they are likely to be deployed and emphasizes user-centered design by addressing the interface between the device and its users. It encourages intuitive and error-free interfaces to enhance usability and focuses on understanding the needs, abilities, and limitations of the intended [40]. Based on these Human Factor Considerations, this section illustrates previously reported PGHD device usability challenges according to the elements of the FDA’s Human Factor Considerations, which are the device use environment, device user, and device user interfaces.

### 2.1. Device Use Environments

Not every consumer wearable or fitness tracker can be used for RPM. The device has to be registered by the FDA as a medical device [41]. While home-use medical devices are defined by the FDA as medical devices that are “intended for users in any environment outside of a professional healthcare facility” [42], wearables are only considered medical devices if the device is “intended for use in the diagnosis of disease or other conditions, or in the cure, mitigation, treatment, or prevention of disease, in man or other animals” [43].

The primary users of home-use medical devices in a virtual environment are consumers, patients, or caregivers, and can be classified as non-clinicians with little technical or health background. Exams can be carried out in an environment that is not monitored by a medical professional, with most likely little governance overseeing the course of a patient’s treatment [44]. Errors may happen during an exam that forces the patient or caregiver to address the issue themselves and figure out if the problem is related to a user error or a technical issue on the device. And errors could cause unsafe device handling and cause patient pain or frustration [44,45,46].

Lyons and Blandford [47] conducted an extensive review of over 600 records of medical device accidents related to infusion pumps in home care settings, uncovering several significant challenges. In-home environments, the absence of continuous monitoring that is typical in clinical settings makes it difficult to promptly identify and address issues. The varying levels of caregiver expertise further complicate the situation, often resulting in under-reported incidents. Technical support is less accessible at home compared to hospitals, causing delays in resolving device malfunctions. Additionally, the process of detecting and accurately reporting incidents is hampered by limited supervision and inconsistent caregiver knowledge. Moreover, the lack of standardized protocols for incident feedback and effective communication among patients, caregivers, and healthcare providers impedes the ability to learn from these incidents and improve safety practices.

Bitterman [48] examined how the patient’s environment impacts the device’s operation. The author illustrated the consequences of the patient’s setting during the exam and the operator’s skills. The author pointed out that the test environment in the healthcare setting is a “standardized, well-regulated, accessible settings, operating under close professional supervision and strict regulations” compared to the patient’s home, which is unique and may result in improper testing procedures and routines. The author also highlighted that the patient will be more motivated and committed to adhering to the test protocol if the home environment is set up to accommodate the required medical exam.

In addition, a healthcare facility is a controlled environment, and exams are governed by a medical professional. Masked hypertension, for instance, is a phenomenon where patients show normal blood pressure while at a healthcare facility, but outside of this controlled environment, blood pressure increases due to various conditions [49]. Baumann et al. [50] researched this further and found that 22% of all participants have concerns that results may be impacted when patients are not in a controlled healthcare environment during an exam.

### 2.2. Device Users

Healthcare is full of challenges, partially due to the intricate nature of the human body and the unique composition of every person. It is, therefore, essential to consider a wide variety of patient characteristics to ensure that usability for PGHD devices is inclusive for all users and patients, regardless of their current health conditions.

Disabled patients who are suffering from health complications, such as respiratory problems or general immobility, may not be able to participate in RPM or virtual care due to their health condition. Dansky et al. [51], for instance, reported that 24% of all patients refused to use a PGHD device with the explanation that they were “too sick to bother”.

Regardless of physical characteristics, there are individual characteristics that every human possesses that will affect usability. Two of the most important ones are health literacy and age. The CDC indicates that only 12% of adults in the United States who make health decisions for themselves and their families have proficient health literacy [52]. This statistic underscores the importance of improving health literacy to empower individuals to make informed choices about their well-being.

Chaniaud et al. [53] investigated the effects of limited device usability on people with different demographics using a medical device (blood pressure and oxygen levels post-surgery). After the participants finished using the device and their exam was completed, every participant’s age, education level, technical knowledge, and health literacy level were categorized and compared, showing that users with little health literacy were outperformed by users who showed a higher comfort level in this area. The researchers concluded with the recommendation for hospitals to always determine the target patient group first to ensure each patient is paired with the proper device, avoiding usability limitations.

To guide clinicians and patients on properly taking an individual’s blood pressure, Murakami & Rakotz [54] and the American Medical Association (AMA) published measurement technique guidelines and how to communicate and document exam results. The article also lists examples of how incorrect device usage and placement can impact the measurement, as shown in Table 1 below.

To show the impact this could have, Campbell et al. [55] studied 69 high-blood-pressure patients while taking blood pressure measurements at their homes without clinical supervision. After the exam, the patient rested for 5 min, followed by a repeated blood pressure exam by a nurse, with the results that the patient’s systolic reading was, on average, 13 mm Hg higher when the exam was performed by the patient, leading to 42% of all blood pressure readings being placed in a different hypertension classification. This illustrates that limited patient knowledge and insufficient measurement techniques by a patient or caregiver can impact the results in a way that could affect a clinician’s treatment plan due to the wrong classification.

In another study, Shin et al. [56] reported qualitative as well as quantitative research findings with a total of 23 Fitbit users who used their device for between 60 and 1073 days. The users’ log revealed two stages: the novelty period and the long-term use period. Based on the study results, the novelty period ended after approximately three months, with 14 participants continuing the device beyond those three months, driven by “personal motivation, social motivation, and gaming motivation”.

### 2.3. Device User Interfaces

User interface (UI) design plays a critical role in the usability and effectiveness of wearables and home-use medical devices. A poorly designed UI can frustrate users, leading them to abandon their devices, hinder data collection, and even lead to safety hazards [57,58], emphasizing the need for wearables to prioritize user experience through clear and engaging UI design.

Reyes et al. [33] reported the results of a usability study with a home-use multi-parameter monitor (a device that measures blood pressure, heart rate, SpO2, respiration rate, and body temperature) to determine whether there were shortcomings in the device based on limited usability. The results showed that 92.3% of all usability problems were related to users being unfamiliar with medical abbreviations provided on the user interface, leading to the incomprehension of measurements. Additionally, 76.6% of the study subjects were unable to interpret the results on the monitor, which is necessary to stay motivated during RPM. Michaud et al. [59] are taking a broader approach and examining various factors that influence patient adherence to RPM protocols. Through a systematic review of existing research, the authors identified key challenges like technical difficulties, user interface complexity, and lack of motivation as barriers to adherence. Additionally, the review highlights the importance of considering factors like age, digital literacy, and socioeconomic status when designing and implementing RPM programs. Addressing these factors can potentially improve patient engagement and adherence, leading to a more effective program.

It is also important to implement user-centered design and listen to the device users, as poor user interface designs can impede usability, including device operation speed, ease of use, and emotional appeal [50,60]. In addition, malfunctioning displays can hinder user interaction and data visualization, which is crucial for operating the device. User interface design should prioritize clarity, simplicity, and intuitive functionality to minimize user frustration and errors [61].

Additionally, a patient’s or caregiver’s motivation and compliance with a treatment protocol can impact the amount and quality of data acquired. Gouveia et al. [62] evaluated patient motivation using a health tracker over a 10-month period with a total of 256 participants. The participants were asked to download an application (app) used for various interactions with a device, such as setting and updating personal goals. They found that 66% of all surveyed patients interacted with the app for more than two days, 38% more than a week, and only 14% above two weeks. The author pointed out that an “app acquisition, in general, is highly exploratory”, but only 69% of apps continue to be used after they have been downloaded. An app is, in many cases, the only way to interact with a device, and if there are flaws in the design, the user may abandon the device [63]. The main drivers for device usage were studied by Asimakopoulos [64]. Thirty-four participants were asked to report their motivation level twice per week over a period of four weeks to determine what drove their motivation to use either a Fitbit or Jawbone fitness tracker. Participants had to answer questions about “reasons for using an activity tracker, motives for choosing their specific tracker, exercise habits, activity tracking and barriers, motivation regarding sustained use, needs and desires, the content impact that prompts motivational behavior, and support for a personalized UX”. The researcher stated that all users were devoted to their devices, with some participants stating that they used the device regularly due to incentives offered by their employers. Furthermore, the results revealed that UX has a direct impact on motivation, with the main drivers being the data displayed and gamification (simplified understanding of data results on the user interface). When tracking usage over time, it is important to also consider the novelty effect. The novelty effect is defined as the “tendency for performance to initially improve when new technology is instituted, not because of any actual improvement in learning or achievement, but in response to increased interest in the new technology” [65]. This includes curiosity about new physical activity data and the technology itself.

## 3. Introducing the RPM Usability Impact Model

While many wearables are able to detect falls and impact automatically, such as a person’s fall off a bicycle [66], home-use medical devices require a user to interact with the device to initiate a measurement, meaning that the user takes an exam by themselves, without medical supervision, and often manually reports the results—therefore the user is the center of their own care.

**Figure 2:**
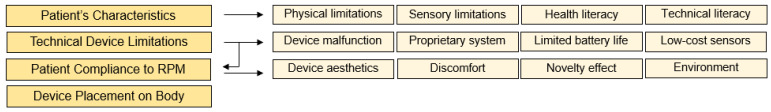
Elements of the RPM Usability Impact model.

Existing research fails, however, to provide a comprehensive framework that illustrates the different types of usability pitfalls that impact medical device usage when health exams and tests are taken outside a medical facility. While the FDA’s existing guidance on human factors and usability engineering [40] provides a strong foundation for medical devices, it leaves out attributes specific to RPM and does not consider when patients and caregivers are by themselves and without clinical supervision when completing an exam in their home. Therefore, this manuscript introduces the RPM Usability Impact model, as displayed in Figure 2, which highlights the difficulties and challenges of generating health data with wearables or home-use medical devices. This model was designed to overcome each of the reported challenges caused by current PGHD devices, providing a guide for manufacturers to develop user-centered devices to fully meet the needs of all patients.

The RPM Usability Impact model (Figure 2) consists of four major pillars and 16 elements and can guide future research for manufacturers to develop wearables and home-use medical devices with proper usability in mind.

The four pillars of this model are defined as patient characteristics, technical device limitations, limited patient compliance, and device placement on the patient’s body. Each of these pillars impacts the accuracy and usability of PGHD devices and is further elaborated on with the review of the literature below.

### 3.1. Patient Characteristics

Every human possesses unique physical and psychological characteristics, which present both opportunities and challenges in healthcare, in particular for the usability of wearables or home-use medical devices.

This begins with the ability to comprehend health data results. As reported in a study by Cutilli & Bennett [67], 14% of U.S. adults possess health literacy skills that fall below a basic level.

Patient engagement with personal health data (PGHD) devices can also be negatively impacted by physical characteristics that make them difficult to use. This is especially true for individuals with mental health conditions, such as anxiety, depression, or autism spectrum disorder [68]. Studies suggest that a significant portion of the population might struggle to adopt telehealth services due to usability [38,69]. Vázquez-de Sebastián et al. [69] further highlight how sensory limitations can hinder the use of mobile health technologies. Often, the root cause of low adherence is a lack of design inclusivity that fails to consider the needs of these user groups.

### 3.2. Technical Device Limitations

Cho et al. [70] recognizes that there has been little research performed in the past on the data quality of PGHD, so the authors used search terms relevant to the topic and categorized 19 articles into “device- and technical-related factors” and “user-related errors” categories. Table 2 below depicts the results, including the amount that appeared in their search.

In a study by Abdolkhani et al. [71], the authors researched which data quality aspects need to be improved during an RPM program and found that health professionals did not consider wearables as a part of disease management because of the many accuracy and quality unknowns of a device driven by the described technical limitations. In this study, all interviewed health professionals were actively involved in RPM programs for diabetes, in particular dealing with continuous glucose monitoring (CGM), cardiac arrhythmia, and sleep disorders. It highlights that participants (all clinicians) in this study are still having too many concerns, mainly about data accuracy or the ability to trust the patient.

The integration of wearable and home-use medical devices into patient treatment plans offers promising opportunities. However, these devices present certain technical limitations that can hinder their effectiveness. Batteries can be depleted, sensors may become loose or misaligned, and wearable straps can break [72]. These breakdowns disrupt data collection and often necessitate device replacement or repair. Also, sweat, dirt, or debris buildup on sensors can interfere with their ability to acquire accurate readings [73]. This highlights the need for improved sensor designs that minimize the impact of external factors on data accuracy.

If a device completely malfunctions, data transfer might be compromised [59]. Robust data transmission protocols are essential to ensure the reliable transfer of patient data. Devices requiring calibration or exhibiting inaccuracy can produce unreliable patient data [74]. Regular calibration practices and the development of self-calibrating devices are crucial for maintaining data accuracy.

### 3.3. Patient Compliance to RPM

The shift towards value-based healthcare, where providers are reimbursed based on patient outcomes [75], emphasizes the importance of patient adherence to using medical devices properly. However, studies reveal concerningly low adherence rates, with factors like demotivation, technical difficulties, and lack of digital literacy playing a significant role [59,76].

To maximize patient engagement and usability, these devices should be designed with minimal user interaction and minimal memorization required for operation, as highlighted by Ronkainen et al. [77]. Additionally, many health conditions necessitate nighttime monitoring, making frequent user interactions impractical. For instance, hypertension requires monitoring throughout the night [78].

The COVID-19 pandemic further amplified the need for non-contact patient monitoring technologies due to the eliminated risk they posed to the spread of the virus [79]. Ideally, these devices should be designed to minimize not just patient contact with the device itself but also be small, flexible, hypoallergenic, and unobtrusive during daily activities, as suggested by research [70,80].

Beyond infection control, other factors impacting adherence include aesthetics and physical comfort. Research by Hsiao & Chen [81] and Yang et al. [82] suggests that a device’s appearance significantly influences user enjoyment and social perception. Additionally, bulky or uncomfortable devices with adhesives or straps may not be suitable for all patients, potentially causing discomfort or allergic reactions. Berg et al. [83] reported that 35% of continuous glucose monitoring users developed skin lesions, and Wong et al. [84] found that nearly half of all users discontinued use due to discomfort and skin reactions. Similarly, Jeffs et al. [85] observed that 32% of patients removed a wearable monitor after discharge from an ICU due to discomfort.

Patient compliance plays a crucial role in the success of remote patient monitoring, and higher levels of patient engagement are associated with improved compliance, contributing to better health outcomes.

### 3.4. Device Placement on Body

Studies highlight various usability challenges when patients or caregivers operate medical devices at home [34,53,86]. For instance, research emphasizes the importance of proper positioning during the measurement of vital signs. Incorrect positioning, like a lack of back support or not having both feet flat on the floor, can significantly alter blood pressure readings, potentially by up to 15 mmHg for systolic readings [87].

Medical devices offer valuable tools for monitoring health and informing treatment decisions. However, the effectiveness of these devices hinges on proper placement for accurate data collection. A recent study focused on the accuracy of wearable pulse oximetry sensors in measuring oxygen saturation. This research examined how placement and skin perfusion (blood flow) affect sensor readings. Improper placement on the finger or inadequate skin perfusion can lead to inaccurate oxygen saturation data [88]. This could have significant consequences for patients with conditions like COPD, where oxygen saturation is a crucial monitoring parameter for treatment decisions. Another study by Sanjo et al. [89] explored the impact of electrode placement on electrocardiogram (ECG) signal quality and diagnostic accuracy. Researchers demonstrated that deviations from the standard electrode placement protocol can alter ECG signal readings. This study also reveals that even well-trained technicians can misplace electrodes by up to 2–3 cm, which can impact the results. This highlights the importance of proper electrode placement for accurate ECG readings and underscores the critical role of proper device placement in ensuring reliable medical data collection.

The introduction of the RPM Usability Impact model should be applied to guide the development of next-generation RPM devices and should be used in conjunction with the FDA’s “Applying human factors and usability engineering to medical devices: guidance for industry and Food and Drug Administration staff” [40]. By understanding the diverse needs and challenges faced by patients in virtual health, such as remote patient monitoring, manufacturers can design more inclusive devices that cater to a wide range of physical, mental, and literacy levels. They should also improve technical robustness and address common technical issues to enhance reliability and ease of use. Manufacturers should incorporate features that minimize user interaction and increase comfort, thereby improving adherence, providing clear instructions, and developing comprehensive user guides and training to ensure proper device placement and operation.

These steps will help ensure that RPM systems are better tailored to individual needs, leading to improved patient outcomes and greater adoption of remote monitoring technologies.

## 4. Discussion

This research introduced the RPM Usability Impact model and described each element and its significance in this emerging market. To afford proper usability for wearables and home-use medical devices, this model will be referenced for future research and by device manufacturers when PGHD devices are defined and manufactured. A lack of compliance with virtual health programs is not because patients are less capable of using these types of devices, but rather, these devices may not have been designed with the users in mind.

The significance of the RPM Usability Impact model lies in its holistic approach to device design and implementation, focusing on the user’s experience and needs. Key impacts include improved health outcomes and a reduction in healthcare costs, yielding several significant outcomes, including future-enhanced device usability, increased patient compliance, accurate data collection, and broader inclusivity. This model also promotes the importance of user-centered design, ensuring that patients and caregivers are included when developing medical devices. Therefore, the definition of a product is the first step in a manufacturing design process when the so-called user-centered design process is applied. The user-centered design describes the phases during a product’s development life cycle, which the International Standard Organization (ISO) defines in ISO 13407 [90] as a four-step process, with the steps outlined as “1. the active involvement of users and clear understanding of user and task requirements; 2. an appropriate allocation of function between user and system; 3. iteration of design solutions; and 4. multi-disciplinary design teams” [91].

This first phase in the user-centered design process requires the manufacturer to gain a deep understanding of a product’s users (such as demographics, profession, or lifestyle choices), the type of product, what the product is used for, the tasks users toned to complete with the product, and the location in which the product is used. To demonstrate the need for manufacturers to apply the RPM Usability Impact model as a frame of reference when defining the product, this first phase uses a case study of a sleep monitoring device to monitor sleep quality for patients with chronic obstructive pulmonary disease (COPD), a condition that causes airflow blockage and breathing-related problems [92]. “Sleep disturbance” is reported as a common symptom by 75% of COPD patients [93], with Divo et al. [94] reporting that the majority of individuals receive a COPD diagnosis above the age of 60 years or older.

This case study represents a 64-year-old retired male. He is a heavy smoker, lives in a rural setting, and is frail due to his medical condition. As a COPD patient, he requires the use of an oxygen tank for most of the day [95], which inhibits many of the activities he enjoys, such as playing golf. He understands the clinical picture of COPD but is reluctant to change. He is also technically challenged and requires many follow-up visits when his doctor makes a change in his course of treatment. He does not have any children and enjoys reading the news if his health allows.

Based on the collected information of this target patient and applying the RPM Usability Impact model, customer needs can be developed that guide the requirements and specifications for the product to be developed. These needs are depicted in the view of the patient, in this case, the 64-year-old male. By applying and aligning with the RPM Usability Impact model, the manufacturer learns that there needs to be importance on the patient’s characteristics, a device’s technical limitation, the patient’s compliance to the treatment protocol (which is also impacted by the device’s technical limitation) and the device placement.

The patient verbatims for this case are listed as customer/patient needs and illustrated in Table 3 below, along with the pillar of the RPM Usability Impact model and the specific element.

There are many wearables and home-use medical devices available that monitor and analyze a patient’s sleep quality and sleep pattern. These devices are worn at a patient’s wrist, around the chest, or require to be placed under a mattress [96]. However, the customer needs in Table 3 and collected, in this case, study illustrate that many COPD patients may not be able to lift a mattress to place a sleep monitoring sensor underneath or will not be able and willing to wear a device attached to their body due to the restrictions they are already experiencing. The 64-old patient’s reluctance to change his habits and technical difficulties necessitate the design of simple, intuitive devices that require minimal user interaction and technical knowledge, and due to his frailty and reliance on an oxygen tank, devices in this case need to be lightweight, portable, and easy to use without requiring significant physical effort. Devices should have an easy-to-navigate interface and straightforward setup process, minimizing the need for technical support, and should be reliable with minimal maintenance requirements.

## 5. Conclusions

This manuscript introduces the RPM Usability Impact model. The model highlights the need for inclusive design, addressing technical limitations, ensuring patient compliance, and proper device placement. Key aspects include ensuring devices are intuitive, easy to use, and reliable and that they fit seamlessly into the patient’s daily life without causing additional burden or complexity.

By integrating user-centered design principles, enhancing training and support, and improving technical robustness, manufacturers can develop RPM devices that are both effective and user-friendly. This approach can help overcome the usability challenges identified and ensure better health outcomes for patients using RPM systems.

While telehealth and RPM, in particular, provide patients with the opportunity to autonomously track their health remotely and transfer data to the provider, specifically for patients in remote areas or patients who are homebound, RPM is a strong contributor to de-personalizing care [97]. Besides the detachment of the patient from their provider, the above collection of articles discusses a large number of challenges that limit and deter a proper diagnosis and treatment due to the potential of a patient’s generated data not being accurate.

RPM has great potential, however, and as Prabhu [98] stated, it could be “the source of medical breakthrough” for more effective patient treatment. With the continued advancement in technology, providing new avenues of data transfer, telehealth, and RPM is here to stay [24,99]. However, PGHD devices will need to afford better usability and the ability to unobtrusively obtain a snapshot of the patient’s health condition.

As the value-based care model continues to be implemented, further driving the adoption of RPM, providers will need better ways to track patient compliance and, with it, patient health outcomes. RPM is, therefore, a promising method that allows a better picture of the patient’s overall health to be obtained, making treatment adjustments between a patient’s visits to a health facility [3]. This will drive continued growth of this market and the adoption of wearables and home-use medical devices by physicians, patients, and consumers, requiring manufacturers to focus on eliminating the elements listed in the RPM Usability Impact model to afford optimal usability and patient experience.

## 6. Future Research

Timothy Chou [100] describes an IoT framework to bring the topic of the Internet of Things closer to the reader. He describes his framework as a method that consists of several elements, which are Things, Connect, Collect, Learn, and Do [100].

PGHD devices are considered Things, and once data collection has started, providers can begin learning from the acquired data and modify a patient’s treatment plan (such as adjusting the medication dose). Collect is the category that is evaluated in this manuscript as the main challenge in RPM programs. However, in order to Learn and Do (such as adjust treatment options), incoming data need to be accurate and reliable, and this starts with the data generation by the patient.

While the value-based care system asks for more data points to make better treatment decisions, better access to data by the providers is also required in the future, such as data integration into e-Health applications. Like RPM, e-Health is a category of telehealth and describes the intersection of health and technology, such as the Electronic Medical Record (EMR) integration into mobile applications [6,101]. Providing patient-generated data in EMRs is crucial so that the data are usable and readily available by patients, caregivers, and providers, and Learn and Do can be executed more effectively, which is necessary for clinicians to use these incoming data without workflow disruption. These incoming data are only worth capturing, however, if the data generation is reliable and known to be accurate. Finally, using Artificial Intelligence (AI) could enhance RPM systems and be integrated within the RPM Usability Impact model to address usability challenges more effectively. For instance, AI can make RPM devices more user-friendly by creating more intuitive interfaces, which aligns with the goals of the RPM Usability Impact model to enhance user experience and provide personalized instructions and feedback, helping users interact with RPM devices more efficiently. AI could also provide immediate alerts to patients and caregivers and predict device failures and maintenance needs, ensuring that RPM devices remain functional and reliable, thus supporting the usability goals of this model. Another AI benefit is the possibility to use the technology to learn from user interactions once enough data have been collected and continuously improve the usability of RPM devices by adapting to user behaviors and preferences over time.

Despite advancements in AI, user-centered design principles embedded in the RPM Usability Impact Model remain crucial. AI can enhance these principles by providing better support and more adaptive systems, but the need to consider human factors in this design still persists.

## Figures and Tables

**Table 1:** Incorrect device usage and placement that account for inaccurate results.

When Patient Has	Blood Pressure Can Change by
Cuff over clothing	10–40 mmHg
Full bladder	10–15 mmHg
Conversation or talking	10–15 mmHg
Unsupported arm	10 mmHg
Unsupported back	5–10 mmHg
Unsupported feet	5–10 mmHg
Crossed legs	2–8 mmHg

**Table 2:** Factors impacting data quality.

User-Related Factors	Amount	Device and Technical Related Factors	Amount
User non-wear	7	Lost bluetooth/network connection	10
Non-wear during battery charging	5	Device Malfunction	8
Misplacement of device on body	5	Low-cost sensors	7
Discomfort of wearing the device	3	Proprietary algorithm or system	6
Forget to wear	2	Software issues	6
Unsatisfied with device appearance	2	Delay in data sync	4
Device not synced by users	1	Quality (accuracy) of algorithm	3

**Table 3:** The need for a sleep monitoring device in COPD patients.

RPM Usability Impact Model		Customer (Patient) Need Description
Pillar	Element	
Patient Characteristics	Physical Limitations	“My frail health does not allow me to lift more than 50 lbs”.
Patient Characteristics	Technical Literacy	“Setting up and connecting the device needs to be simple; I can barely check my email”.
Technical Device Limitations	Limited Battery Life	“I do not want to keep charging my battery for all these devices. It is difficult for me, and once it is fully charged, I often forget about them”.
Patient Compliance to RPM	Novelty effect	“I have used a Fitbit before to track steps, but after 30 days I stopped using it. When my doctor encouraged me to use to track my steps, I wore it again for a week or two”.
Patient Compliance to RPM	Discomfort	“I have an oxygen tank with me most of the day; there’s no way I will attach anything else to my body since I am already so restricted”.
Device Placement on Body	N/A	“I have used a respiratory belt before to monitor my respiratory rate, but I never knew exactly how tight it should be”.

## References

[1] Grand View Research Wearable Medical Devices Market Size Report 2021–2028. https://www.grandviewresearch.com/industry-analysis/wearable-medical-devices-market.

[2] McCarthy J. One in Five U.S. Adults Use Health Apps, Wearable Trackers. https://news.gallup.com/poll/269096/one-five-adults-health-apps-wearable-trackers.aspx.

[3] The Healthcare Information and Management Systems Society (2018). Healthcare Coaching: Multiplying the Value of Wearables and Patient-Generated Health Data.

[4] Tull M.T., Edmonds K.A., Scamaldo K.M., Richmond J.R., Rose J.P., Gratz K.L. (2020). Psychological Outcomes Associated with Stay-at-Home Orders and the Perceived Impact of COVID-19 on Daily Life. Psychiatry Res..

[5] Bestsennyy O., Gilbert G., Harris A., Rost J. (2020). Telehealth: A Quarter-Trillion-Dollar Post-COVID-19 Reality.

[6] Nejm Catalyst What Is Telehealth?. https://catalyst.nejm.org/doi/full/10.1056/CAT.18.0268.

[7] Abubakar A., Sinclair J. (2020). The Emerging Role of Community Pharmacists in Remote Patient Monitoring Services. Pharmacy.

[8] The National Telehealth Policy Center What Is Telehealth?-CCHP. https://www.cchpca.org/what-is-telehealth/?category=mobile-health.

[9] Vegesna A., Tran M., Angelaccio M., Arcona S. (2017). Remote patient monitoring via non-invasive digital technologies: A systematic review. Telemed. e-Health.

[10] Peretz D., Arnaert A., Ponzoni N.N. (2018). Determining the cost of implementing and operating a remote patient monitoring programme for the elderly with chronic conditions: A systematic review of economic evaluations. J. Telemed. Telecare.

[11] Morrissey J. (2014). Remote patient monitoring: How mobile devices will curb chronic conditions. Med. Econ..

[12] Maciejewski M., Surtel W., Wójcik W., Masiak J., Dzida G., Horoch A. (2014). Telemedical systems for home monitoring of patients with chronic conditions in rural environment. Ann. Agric. Environ. Med..

[13] Nakamura N., Koga T., Iseki H. (2014). A meta-analysis of remote patient monitoring for chronic heart failure patients. J Telemed. Telecare.

[14] Centers for Disease Control and Prevention Health and Economic Costs of Chronic Diseases. https://www.cdc.gov/pcd/issues/2024/23_0267.htm.

[15] Tang M., Nakamoto C.H., Stern A.D., Mehrotra A. (2022). Trends in Remote Patient Monitoring Use in Traditional Medicare. JAMA Intern. Med..

[16] Mueller P.S. (2022). Trends in Use of Remote Patient Monitoring. NEJM J. Watch..

[17] Weigel A.R.G. Opportunities and Barriers for Telemedicine in the U.S. during the COVID-19 Emergency and Beyond. https://www.kff.org/womens-health-policy/issue-brief/opportunities-and-barriers-for-telemedicine-in-the-u-s-during-the-covid-19-emergency-and-beyond/.

[18] Olmos C., Franco E., Suárez-Barrientos A., Fortuny E., Martín-García A., Viliani D., Macaya C., de Isla L.P. (2014). Wearable wireless remote monitoring system: An alternative for prolonged electrocardiographic monitoring. Int. J. Cardiol..

[19] Prabhu S. The Real Value of IoT at Home. Healthcare Innovation. https://www.hcinnovationgroup.com/interoperability-hie/article/13007936/the-real-value-of-iot-at-home.

[20] Freitag T.B., Taylor G., Wick L., Cunningham J., Alexy T. (2019). Novel remote patient monitoring system improves key outcomes. J. Card. Fail..

[21] Siwicki B. How Remote Patient Monitoring is Moving into the Mainstream. Healthcare IT News. https://www.healthcareitnews.com/news/how-remote-patient-monitoring-moving-mainstream.

[22] Ong W.L., Schouwenburg M.G., Van Bommel A.C., Stowell C., Allison K.H., Benn K.E., Browne J.P., Cooter R.D., Delaney G.P., Duhoux F.P. (2017). A standard set of value-based patient-centered outcomes for breast cancer: The international consortium for health outcomes measurement (ICHOM) initiative. JAMA Oncol..

[23] Dey P., Jarrin R., Mori M., Geirsson A., Krumholz H.M. (2021). Leveraging Remote Physiologic Monitoring in the COVID-19 Pandemic to Improve Care after Cardiovascular Hospitalizations. Circ. Cardiovasc. Qual. Outcomes.

[24] Roblyer D.M. (2020). Perspective on the increasing role of optical wearables and remote patient monitoring in the COVID-19 era and beyond. J. Biomed. Opt..

[25] Tashnek D. CMS Announces 3 Significant Corrections to Remote Patient Monitoring. Becker’s Hospital Review. https://www.beckershospitalreview.com/telehealth/cms-announces-3-significant-corrections-to-remote-patient-monitoring.html.

[26] Strategic Market Research Global Remote Patient Monitoring Statistics 2023-Facts & Figures. Strategic Market Research. https://www.strategicmarketresearch.com/blogs/remote-patient-monitoring-statistics.

[27] Wicklund E. CMS Proposes Significant Changes to Remote Patient Monitoring Coverage. mHealth Intelligence. https://mhealthintelligence.com/news/cms-proposes-significant-changes-to-remote-patient-monitoring-coverage.

[28] Aalam A.A., Hood C., Donelan C., Rutenberg A., Kane E.M., Sikka N. (2021). Remote patient monitoring for ED discharges in the COVID-19 pandemic. Emerg. Med. J..

[29] Zarola T. ‘Home’ Is Where the Heart Is. https://www.analog.com/en/resources/technical-articles/home-is-where-the-heart-is.html.

[30] Noah B., Keller M.S., Mosadeghi S., Stein L., Johl S., Delshad S., Tashjian V.C., Lew D., Kwan J.T., Jusufagic A. (2018). Impact of remote patient monitoring on clinical outcomes: An updated meta-analysis of randomized controlled trials. NPJ Digit. Med..

[31] Baumann S., Stone R., Abdelall E., Srikrishnan V., Schnieders T., Fales C., Mumani A. (2019). Implementing Blockchain to Enhance Usability of Patient-Generated Data. Proceedings of the Human Factors and Ergonomics Society Annual Meeting.

[32] Jokela T. Case Studies on a Quality Model based on the ISO 9241-11 Definition of Usability. Proceedings of the International COST294 Workshop on User Interface Quality Models.

[33] Reyes P., Larée D., Weinstein A., Jara Á. (2018). Towards a conceptual model for the use of home healthcare medical devices: The multi-parameter monitor case. PLoS ONE.

[34] Cifter A.S. (2017). Blood pressure monitor usability problems detected through human factors evaluation. Ergon. Des..

[35] Kortum P., Peres S.C. (2015). Evaluation of home health care devices: Remote usability assessment. JMIR Hum. Factors.

[36] Zhang J., Johnson T.R., Patel V.L., Paige D.L., Kubose T. (2003). Using usability heuristics to evaluate patient safety of medical devices. J. Biomed. Inform..

[37] Furniss D., Masci P., Curzon P., Mayer A., Blandford A. (2014). 7 Themes for guiding situated ergonomic assessments of medical devices: A case study of an inpatient glucometer. Appl. Ergon..

[38] Rodríguez-Fernández J.M., Danies E., Hoertel N., Galanter W., Saner H., Franco O.H. (2022). Telemedicine Readiness Across Medical Conditions in a US National Representative Sample of Older Adults. J. Appl. Gerontol..

[39] Baumann S., Stone R., Kim J.Y.-M. (2024). Introducing the Pi-CON Methodology to Overcome Usability Deficits during Remote Patient Monitoring. Sensors.

[40] U.S. Department of Health and Human Services, Food and Drug Administration (2016). Applying Human Factors and Usability Engineering to Medical Devices.

[41] Lacktman N.M. 2021 Medicare Remote Patient Monitoring FAQs: CMS Issues Final Rule Health Care Law Today. https://www.foley.com/en/insights/publications/2020/12/2021-remote-patient-monitoring-cms-final-rule.

[42] U.S. Food & Drug Administration Home Use Devices. https://www.fda.gov/medical-devices/home-health-and-consumer-devices/home-use-devices.

[43] U.S. Food & Drug Administration (2019). How to Determine If Your Product Is a Medical Device. https://www.fda.gov/medical-devices/classify-your-medical-device/how-determine-if-your-product-medical-device.

[44] Tase A., Vadhwana B., Buckle P., Hanna G.B. (2022). Usability challenges in the use of medical devices in the home environment: A systematic review of literature. Appl. Ergon..

[45] Rajkomar A., Mayer A., Blandford A. (2015). Understanding safety–critical interactions with a home medical device through Distributed Cognition. J. Biomed. Inform..

[46] Goodman-Deane J., Ward J., Hosking I., Clarkson P.J. (2014). A comparison of methods currently used in inclusive design. Appl. Ergon..

[47] Lyons I., Blandford A. (2018). Safer healthcare at home: Detecting, correcting and learning from incidents involving infusion devices. Appl. Ergon..

[48] Bitterman N. (2011). Design of medical devices—A home perspective. Eur. J. Intern. Med..

[49] Pickering T.G. (1991). Clinical applications of ambulatory blood pressure monitoring: The white coat syndrome. Clin. Investig. Med..

[50] Baumann S., Stone R.T., Genschel U., Mgaedeh F. (2023). The Pi-CON Methodology Applied: Operator Errors and Preference Tracking of a Novel Ubiquitous Vital Signs Sensor and Its User Interface. Int. J. Hum. Comput. Interact..

[51] Dansky K.H., Bowles K.H., Palmer L. (1999). How telehomecare affects patients. Caring.

[52] CDC Health Literacy: Accurate, Accessible and Actionable Health Information for All. https://www.cdc.gov/healthliteracy/index.html.

[53] Chaniaud N., Megalakaki O., Capo S., Loup-Escande E. (2021). Effects of User Characteristics on the Usability of a Home-Connected Medical Device (Smart Angel) for Ambulatory Monitoring: Usability Study. JMIR Hum. Factors.

[54] Murakami L., Rakotz M. (2015). Self-measured Blood Pressure Monitoring Program: Engaging Patients in Self-Measurement. Improving Health Outcomes: Blood Pressure. https://www.ama-assn.org/sites/ama-assn.org/files/corp/media-browser/public/about-ama/iho-bp-engaging-patients-in-self-measurment_0.pdf.

[55] Campbell N.R.C., Milkovich L., Burgess E., McKay D.W. (2001). Self-measurement of blood pressure: Accuracy, patient preparation for readings, technique and equipment. Blood Press. Monit..

[56] Shin G., Feng Y., Jarrahi M.H., Gafinowitz N. (2019). Beyond novelty effect: A mixed-methods exploration into the motivation for long-term activity tracker use. JAMIA Open.

[57] Khinvasara T., Ness S., Tzenios N. (2023). Risk Management in Medical Device Industry. J. Eng. Res. Rep..

[58] Nguyen C.C., Wong S., Thai M.T., Hoang T.T., Phan P.T., Davies J., Wu L., Tsai D., Phan H.P., Lovell N.H. (2023). Advanced user interfaces for teleoperated surgical robotic systems. Adv. Sens. Res..

[59] Michaud A., Vadeboncoeur A., Cloutier L., Goupil R. (2021). The feasibility of home self-assessment of vital signs and symptoms: A new key to telehealth for individuals?. Int. J. Med. Inform..

[60] Minnesota Diversified Industries Understanding User Needs When Designing Medical Devices. Minnesota Diversified Industries. https://www.mdi.org/blog/post/understanding-user-needs-when-designing-medical-devices/.

[61] Cox A., Gould S., Cecchinato M., Iacovides I., Renfree I. Design Frictions for Mindful Interactions: The Case for Microboundaries. Proceedings of the 2016 CHI Conference Extended Abstracts on Human Factors in Computing Systems.

[62] Gouveia R., Karapanos E., Hassenzahl M. How do we engage with activity trackers? A longitudinal study of Habito. Proceedings of the 2015 ACM International Joint Conference on Pervasive and Ubiquitous Computing.

[63] Georgescu M., Strainu R. (2016). The Importance of Natural User Interface in Designing Mobile Learning Apps. The International Scientific Conference eLearning and Software for Education.

[64] Asimakopoulos S., Asimakopoulos G., Spillers F. (2017). Motivation and user engagement in fitness tracking: Heuristics for mobile healthcare wearables. Informatics.

[65] Ngai J. Designing with Data. Interpreting and Analyzing Data as a Designer. UX Collective. https://uxdesign.cc/designing-with-data-ed721ffa008e.

[66] Brown J. Bike Crash Left Spokane Man Unconscious, so His Apple Watch Called 911. The Seattle Times. https://www.seattletimes.com/seattle-news/bike-crash-left-spokane-man-unconscious-but-his-apple-watch-called-911/.

[67] Cutilli C.C., Bennett I.M. (2009). Understanding the health literacy of America results of the national assessment of adult literacy. Orthop. Nurs./Natl. Assoc. Orthop. Nurses.

[68] Damian A.J., Stinchfield K., Kearney R.T. (2022). Telehealth and beyond: Promoting the mental well-being of children and adolescents during COVID. Front. Pediatr..

[69] Vázquez-de Sebastián J., Ciudin A., Castellano-Tejedor C. (2021). Analysis of Effectiveness and Psychological Techniques Implemented in mHealth Solutions for Middle-Aged and Elderly Adults with Type 2 Diabetes: A Narrative Review of the Literature. J. Clin. Med..

[70] Cho S., Ensari I., Weng C., Kahn M.G., Natarajan K. (2021). Factors Affecting the Quality of Person-Generated Wearable Device Data and Associated Challenges: Rapid Systematic Review. JMIR Mhealth Uhealth.

[71] Abdolkhani R., Gray K., Borda A., DeSouza R. (2019). Patient-generated health data management and quality challenges in remote patient monitoring. JAMIA Open.

[72] Heikenfeld J., Jajack A., Rogers J., Gutruf P., Tian L., Pan T., Li R., Khine M., Kim J., Wang J. (2018). Wearable sensors: Modalities, challenges, and prospects. Lab Chip.

[73] Shei R.-J., Holder I.G., Oumsang A.S., Paris B.A., Paris H.L. (2022). Wearable activity trackers–advanced technology or advanced marketing?. Eur. J. Appl. Physiol..

[74] Wang Z., Fang D., Liu X., Zhang L., Duan H., Wang C., Guo K. (2023). Consumer acceptance of sports wearables: The role of products attributes. Sage Open.

[75] Novikov D., Cizmic Z., Feng J.E., Iorio R., Meftah M. (2018). The historical development of value-based care: How we got here. JBJS.

[76] Motolese F., Magliozzi A., Puttini F., Rossi M., Capone F., Karlinski K., Stark-Inbar A., Yekutieli Z., Di Lazzaro V., Marano M. (2020). Parkinson’s disease remote patient monitoring during the COVID-19 lockdown. Front. Neurol..

[77] Ronkainen S., Koskinen E., Liu Y., Korhonen P. (2010). Environment analysis as a basis for designing multimodal and multidevice user interfaces. Hum. Comput. Interact..

[78] Lurbe E., Sorof J.M., Daniels S.R. (2004). Clinical and research aspects of ambulatory blood pressure monitoring in children. J. Pediatr..

[79] Khan M.B., Zhang Z., Li L., Zhao W., Hababi M.A., Yang X., Abbasi Q.H. (2020). A systematic review of non-contact sensing for developing a platform to contain COVID-19. Micromachines.

[80] Majumder S., Mondal T., Deen M.J. (2017). Wearable sensors for remote health monitoring. Sensors.

[81] Hsiao K.-L., Chen C.-C. (2018). What drives smartwatch purchase intention? Perspectives from hardware, software, design, and value. Telemat. Inform..

[82] Yang H., Yu J., Zo H., Choi M. (2016). User acceptance of wearable devices: An extended perspective of perceived value. Telemat. Inform..

[83] Berg A.K., Simonsen A.B., Svensson J. (2018). Perception and possible causes of skin problems to insulin pump and glucose sensor: Results from pediatric focus groups. Diabetes Technol. Ther..

[84] Wong J.C., Foster N.C., Maahs D.M., Raghinaru D., Bergenstal R.M., Ahmann A.J., Peters A.L., Bode B.W., Aleppo G., Hirsch I.B. (2014). Real-time continuous glucose monitoring among participants in the T1D Exchange clinic registry. Diabetes Care.

[85] Jeffs E., Vollam S., Young J.D., Horsington L., Lynch B., Watkinson P.J. (2016). Wearable monitors for patients following discharge from an intensive care unit: Practical lessons learnt from an observational study. J. Adv. Nurs..

[86] Jia Y., Wang W., Wen D., Liang L., Gao L., Lei J. (2018). Perceived user preferences and usability evaluation of mainstream wearable devices for health monitoring. PeerJ.

[87] Muntner P., Shimbo D., Carey R.M., Charleston J.B., Gaillard T., Misra S., Myers M.G., Ogedegbe G., Schwartz J.E., Townsend R.R. (2019). Measurement of blood pressure in humans: A scientific statement from the American Heart Association. Hypertension.

[88] Knight S., Lipoth J., Namvari M., Gu C., Hedayati M., Syed-Abdul S., Spiteri R.J. (2022). The Accuracy of Wearable Photoplethysmography Sensors for Telehealth Monitoring: A Scoping Review. Telemed. e-Health.

[89] Sanjo K., Hebiguchi K., Tang C., Rashed E.A., Kodera S., Togo H., Hirata A. (2024). Sensitivity of Electrocardiogram on Electrode-Pair Locations for Wearable Devices: Computational Analysis of Amplitude and Waveform Distortion. Biosensors.

[90] (1999). Human-Centred Design Processes for Interactive Systems.

[91] Devi K.R., Sen A.M., Hemachandran K. (2014). A working Framework for the User-Centered Design Approach and a Survey of the available Methods. https://api.semanticscholar.org/CorpusID:17792363.

[92] Raherison C., Girodet P.-O. (2009). Epidemiology of COPD. Eur. Respir. Rev..

[93] Agusti A., Hedner J., Marin J., Barbé F., Cazzola M., Rennard S. (2011). Night-time symptoms: A forgotten dimension of COPD. Eur. Respir. Rev..

[94] Divo M., Marin J.M., Casanova C., Cabrera Lopez C., Pinto-Plata V.M., Marin-Oto M., Polverino F., de-Torres J.P., Billheimer D., Celli B.R. (2022). Comorbidities and mortality risk in adults younger than 50 years of age with chronic obstructive pulmonary disease. Respir. Res..

[95] Cullen D. (2006). Long term oxygen therapy adherence and COPD: What we don’t know. Chron Respir. Dis..

[96] Kelly J., Strecker R., Bianchi M. (2012). Recent Developments in Home Sleep-Monitoring Devices. ISRN Neurol..

[97] Sharma U., Clarke M. (2014). ‘Nurses’ and community support workers’ experience of telehealth: A longitudinal case study. BMC Health Serv. Res..

[98] Prabhu S.R. The Real Value of IoT at Home. Health Management Technology. http://search.ebscohost.com/login.aspx?direct=true&db=cin20&AN=117941286&site=ehost-live.

[99] Kaye R., Rosen-Zvi M., Ron R. (2020). Digitally-enabled remote care for cancer patients: Here to stay. Seminars in Oncology Nursing.

[100] Chou T. (2017). Precision-Principles, Practices and Solutions for the Internet of Things.

[101] Femi A.G., Musa Ali A., Williams Enam J. (2023). E-Health: A Comprehensive Overview. EasyChair Prepr..

